# 3-Ethyl-4-methyl-1*H*-pyrazol-2-ium-5-olate

**DOI:** 10.1107/S160053681102808X

**Published:** 2011-07-23

**Authors:** R. S. Rathore, T. Narasimhamurthy, R. Venkat Ragavan, V. Vijayakumar, S. Sarveswari

**Affiliations:** a Bioinformatics Infrastructure Facility, School of Life Science, University of Hyderabad, Hyderabad 500 046, India; b Materials Research Center, Indian Institute of Science, Bangalore 560 012, India; c Organic Chemistry Division, School of Advanced Sciences, VIT University, Vellore 632 014, India

## Abstract

The title compound, C_6_H_10_N_2_O, is a zwitterionic pyrazole derivative. The crystal packing is predominantly governed by a three-center iminium–amine N^+^—H⋯O^−^⋯H—N inter­action, leading to an undulating sheet-like structure lying parallel to (100).

## Related literature

For related structures and the preparation of similar compounds, see: Ragavan *et al.* (2009 ▶, 2010 ▶) and references therein. For related salt-bridge-mediated sheet structures, see: Shylaja *et al.* (2008 ▶).
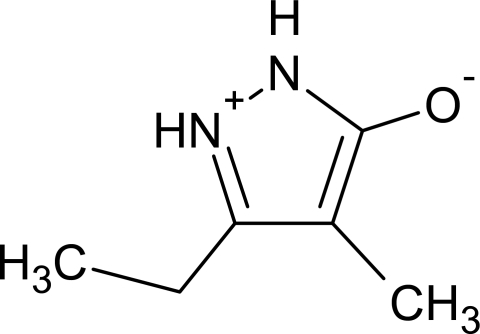


## Experimental

### 

#### Crystal data

C_6_H_10_N_2_O
*M* *_r_* = 126.16
Monoclinic, 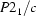

*a* = 9.1299 (15) Å
*b* = 7.1600 (11) Å
*c* = 11.374 (2) Å
β = 113.232 (9)°
*V* = 683.2 (2) Å^3^
*Z* = 4
Mo *K*α radiation
μ = 0.09 mm^−1^
*T* = 296 K
0.21 × 0.19 × 0.11 mm

#### Data collection

Bruker APEXII CCD diffractometer
Absorption correction: multi-scan (*SADABS*; Bruker, 2001 ▶) *T*_min_ = 0.64, *T*_max_ = 0.83
12120 measured reflections
1332 independent reflections
961 reflections with *I* > 2σ(*I*)
*R*_int_ = 0.034

#### Refinement

*R*[*F* ^2^ > 2σ(*F* ^2^)] = 0.049
*wR*(*F* ^2^) = 0.136
*S* = 1.03
1332 reflections
92 parameters
H atoms treated by a mixture of independent and constrained refinement
Δρ_max_ = 0.17 e Å^−3^
Δρ_min_ = −0.21 e Å^−3^

### 

Data collection: *APEX2* (Bruker, 2007 ▶); cell refinement: *SAINT-Plus* (Bruker, 2007 ▶); data reduction: *SAINT-Plus*; program(s) used to solve structure: *SHELXS97* (Sheldrick, 2008 ▶); program(s) used to refine structure: *SHELXL97* (Sheldrick, 2008 ▶); molecular graphics: *ORTEP-3* (Farrugia, 1997 ▶) and *PLATON* (Spek, 2009 ▶); software used to prepare material for publication: *SHELXL97* and *PLATON* (Spek, 2009 ▶).

## Supplementary Material

Crystal structure: contains datablock(s) global, I. DOI: 10.1107/S160053681102808X/su2287sup1.cif

Structure factors: contains datablock(s) I. DOI: 10.1107/S160053681102808X/su2287Isup2.hkl

Supplementary material file. DOI: 10.1107/S160053681102808X/su2287Isup3.cml

Additional supplementary materials:  crystallographic information; 3D view; checkCIF report

## Figures and Tables

**Table 1:** Hydrogen-bond geometry (Å, °)

*D*—H⋯*A*	*D*—H	H⋯*A*	*D*⋯*A*	*D*—H⋯*A*
N1—H1⋯O5^i^	0.91 (2)	1.82 (2)	2.730 (2)	175 (2)
N2—H2⋯O5^ii^	0.96 (2)	1.75 (2)	2.693 (2)	168 (2)

Symmetry codes: (i) 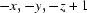; (ii) 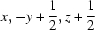.

**Fig. 1.**
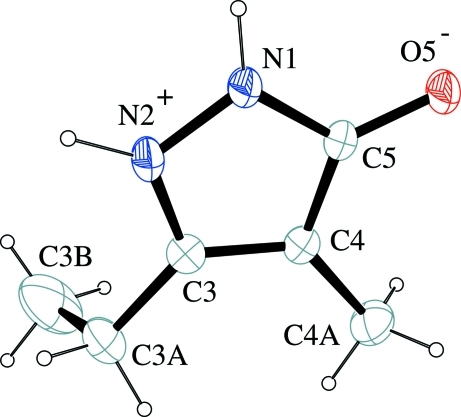
A view of the molecular structure of the title molecule, with labelling scheme and displacement ellipsoids drawn at the 30% probability level.

**Fig. 2.**
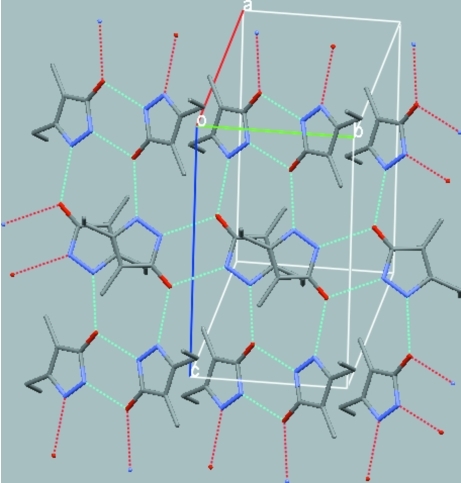
A view of the N—H···O hydrogen bonded (dashed cyan lines) sheet structure in the crystal structure of the title compound (see Table 1 for details).

## supplementary crystallographic information

### Comment

As a part of our interest in antimicrobial compounds, we have synthesized the title pyrazole derivative using the procedure described earlier by (Ragavan *et al.*, 2009, and references therein; 2010, and references therein).

The molecular structure of the title molecule is shown in Fig 1. The methyl atom (C3B) of the 3-ethyl substituent lies out of the mean plane of the pyrazole moiety (N1,N2,C3-C5) by 1.366 (4) Å.

The crystal packing is a fine balance of strong N—H···O hydrogen bonds (Table 1) and salt bridges, which normally tend to promote the formation of a planar structure and compact packing (Shylaja *et al.*, 2008). In the title compound all the hydrogen bonding donors, iminium N^+^H (N1) and amine NH (N2), and the O^-^(O1) acceptor, are in the plane of the pyrazole moiety, which would normally yield a planar hydrogen-bonded structure. However, in order to accommodate the out-of-plane methyl group, (C3B), an undulating hydrogen bonded sheet-like structure, lieing paralallel to (100), is formed (Fig. 2).

### Experimental

The title compound was synthesized using the method described earlier by (Ragavan *et al.*, 2009, 2010). It was crystallized using an ethanol-chloroform (1:1) mixture. Yield, 74%; m.p. 779-780 K.

### Refinement

The NH atoms were located in a difference Fourier map and were freely refined: N2—H2 = 0.92 (2) Å and N1^+^—H1 = 0.95 (3) Å. The methylene and methyl hydrogen atoms were placed in calculated positions and refined as riding atoms: C-H = 0.97 and 0.96 Å, for CH and CH_3_ H-atoms, respectively, with U_iso_(H) = k × U_eq_(C,) where k = 1.5 for CH_3_ H-atoms and 1.2 for the CH H-atoms.

### Crystal data

**Table:**

C_6_H_10_N_2_O	*F*(000) = 272
*M**_r_* = 126.16	*D*_x_ = 1.227 Mg m^−^^3^
Monoclinic, *P*2_1_/*c*	Mo *K*α radiation, λ = 0.71073 Å
Hall symbol: -P 2ybc	Cell parameters from 3015 reflections
*a* = 9.1299 (15) Å	θ = 2.4–22.9°
*b* = 7.1600 (11) Å	µ = 0.09 mm^−^^1^
*c* = 11.374 (2) Å	*T* = 296 K
β = 113.232 (9)°	Plate, colourless
*V* = 683.2 (2) Å^3^	0.21 × 0.19 × 0.11 mm
*Z* = 4	

### Data collection

**Table:**

Bruker APEXII CCD diffractometer	1332 independent reflections
Radiation source: fine-focus sealed tube	961 reflections with *I* > 2σ(*I*)
graphite	*R*_int_ = 0.034
φ and ω scans	θ_max_ = 26.0°, θ_min_ = 2.4°
Absorption correction: multi-scan (*SADABS*; Bruker, 2001)	*h* = −11→11
*T*_min_ = 0.64, *T*_max_ = 0.83	*k* = −8→8
12120 measured reflections	*l* = −13→13

### Refinement

**Table:**

Refinement on *F*^2^	Primary atom site location: structure-invariant direct methods
Least-squares matrix: full	Secondary atom site location: difference Fourier map
*R*[*F*^2^ > 2σ(*F*^2^)] = 0.049	Hydrogen site location: inferred from neighbouring sites
*wR*(*F*^2^) = 0.136	H atoms treated by a mixture of independent and constrained refinement
*S* = 1.03	*w* = 1/[σ^2^(*F*_o_^2^) + (0.0674*P*)^2^ + 0.2195*P*] where *P* = (*F*_o_^2^ + 2*F*_c_^2^)/3
1332 reflections	(Δ/σ)_max_ < 0.001
92 parameters	Δρ_max_ = 0.17 e Å^−^^3^
0 restraints	Δρ_min_ = −0.21 e Å^−^^3^

### Special details

**Table:**

Geometry. All e.s.d.'s (except the e.s.d. in the dihedral angle between two l.s. planes) are estimated using the full covariance matrix. The cell e.s.d.'s are taken into account individually in the estimation of e.s.d.'s in distances, angles and torsion angles; correlations between e.s.d.'s in cell parameters are only used when they are defined by crystal symmetry. An approximate (isotropic) treatment of cell e.s.d.'s is used for estimating e.s.d.'s involving l.s. planes.
Refinement. Refinement of *F*^2^ against ALL reflections. The weighted *R*-factor *wR* and goodness of fit *S* are based on *F*^2^, conventional *R*-factors *R* are based on *F*, with *F* set to zero for negative *F*^2^. The threshold expression of *F*^2^ > σ(*F*^2^) is used only for calculating *R*-factors(gt) *etc*. and is not relevant to the choice of reflections for refinement. *R*-factors based on *F*^2^ are statistically about twice as large as those based on *F*, and *R*- factors based on ALL data will be even larger.

### Fractional atomic coordinates and isotropic or equivalent isotropic displacement parameters (Å^2^)

**Table:**

	*x*	*y*	*z*	*U*_iso_*/*U*_eq_	
N1	0.0756 (2)	0.1929 (2)	0.58699 (14)	0.0427 (5)	
H1	0.029 (3)	0.087 (3)	0.601 (2)	0.051 (6)*	
N2	0.1373 (2)	0.3275 (2)	0.67795 (15)	0.0462 (5)	
H2	0.116 (3)	0.326 (3)	0.754 (2)	0.062 (6)*	
O5	0.07244 (17)	0.12652 (19)	0.38750 (11)	0.0489 (4)	
C3	0.2107 (2)	0.4552 (3)	0.63369 (17)	0.0402 (5)	
C3A	0.2903 (3)	0.6189 (3)	0.7141 (2)	0.0573 (6)	
H3A1	0.2994	0.7177	0.6591	0.069*	
H3A2	0.2238	0.6648	0.7566	0.069*	
C3B	0.4512 (4)	0.5766 (4)	0.8123 (3)	0.1014 (12)	
H3B1	0.4424	0.4859	0.8714	0.152*	
H3B2	0.4982	0.6889	0.8577	0.152*	
H3B3	0.5171	0.5277	0.7714	0.152*	
C4	0.1999 (2)	0.4015 (2)	0.51474 (17)	0.0367 (5)	
C4A	0.2643 (3)	0.4995 (3)	0.4291 (2)	0.0544 (6)	
H41	0.3131	0.6149	0.4681	0.082*	
H42	0.1789	0.5248	0.3482	0.082*	
H43	0.3422	0.4216	0.4162	0.082*	
C5	0.1135 (2)	0.2330 (3)	0.48611 (16)	0.0359 (5)	

### Atomic displacement parameters (Å^2^)

**Table:**

	*U*^11^	*U*^22^	*U*^33^	*U*^12^	*U*^13^	*U*^23^
N1	0.0661 (11)	0.0389 (9)	0.0308 (8)	−0.0145 (8)	0.0275 (8)	−0.0061 (7)
N2	0.0698 (12)	0.0444 (10)	0.0313 (9)	−0.0109 (8)	0.0275 (8)	−0.0098 (7)
O5	0.0759 (10)	0.0484 (8)	0.0304 (7)	−0.0197 (7)	0.0295 (7)	−0.0092 (6)
C3	0.0470 (11)	0.0362 (10)	0.0369 (10)	−0.0008 (8)	0.0162 (9)	−0.0008 (8)
C3A	0.0725 (15)	0.0467 (12)	0.0519 (13)	−0.0103 (11)	0.0237 (12)	−0.0149 (10)
C3B	0.084 (2)	0.082 (2)	0.097 (2)	−0.0121 (16)	−0.0077 (17)	−0.0341 (18)
C4	0.0428 (10)	0.0361 (10)	0.0322 (9)	−0.0018 (8)	0.0159 (8)	0.0016 (8)
C4A	0.0632 (14)	0.0566 (13)	0.0485 (12)	−0.0143 (11)	0.0274 (11)	0.0035 (10)
C5	0.0455 (10)	0.0378 (10)	0.0266 (9)	−0.0018 (8)	0.0165 (8)	0.0003 (8)

### Geometric parameters (Å, °)

**Table:**

N1—C5	1.354 (2)	C3A—H3A2	0.9700
N1—N2	1.363 (2)	C3B—H3B1	0.9600
N1—H1	0.92 (2)	C3B—H3B2	0.9600
N2—C3	1.343 (3)	C3B—H3B3	0.9600
N2—H2	0.95 (3)	C4—C5	1.408 (3)
O5—C5	1.284 (2)	C4—C4A	1.495 (3)
C3—C4	1.372 (3)	C4A—H41	0.9600
C3—C3A	1.488 (3)	C4A—H42	0.9600
C3A—C3B	1.484 (4)	C4A—H43	0.9600
C3A—H3A1	0.9700		
			
C5—N1—N2	109.01 (16)	H3B1—C3B—H3B2	109.5
C5—N1—H1	128.2 (13)	C3A—C3B—H3B3	109.5
N2—N1—H1	122.3 (13)	H3B1—C3B—H3B3	109.5
C3—N2—N1	108.38 (16)	H3B2—C3B—H3B3	109.5
C3—N2—H2	130.8 (14)	C3—C4—C5	106.50 (16)
N1—N2—H2	120.5 (14)	C3—C4—C4A	128.08 (17)
N2—C3—C4	109.04 (16)	C5—C4—C4A	125.42 (17)
N2—C3—C3A	120.03 (18)	C4—C4A—H41	109.5
C4—C3—C3A	130.90 (18)	C4—C4A—H42	109.5
C3B—C3A—C3	113.6 (2)	H41—C4A—H42	109.5
C3B—C3A—H3A1	108.8	C4—C4A—H43	109.5
C3—C3A—H3A1	108.8	H41—C4A—H43	109.5
C3B—C3A—H3A2	108.8	H42—C4A—H43	109.5
C3—C3A—H3A2	108.8	O5—C5—N1	122.03 (16)
H3A1—C3A—H3A2	107.7	O5—C5—C4	130.92 (17)
C3A—C3B—H3B1	109.5	N1—C5—C4	107.05 (15)
C3A—C3B—H3B2	109.5		
			
C5—N1—N2—C3	1.6 (2)	C3A—C3—C4—C4A	−1.7 (3)
N1—N2—C3—C4	−1.4 (2)	N2—N1—C5—O5	178.68 (17)
N1—N2—C3—C3A	−179.69 (17)	N2—N1—C5—C4	−1.2 (2)
N2—C3—C3A—C3B	80.6 (3)	C3—C4—C5—O5	−179.5 (2)
C4—C3—C3A—C3B	−97.3 (3)	C4A—C4—C5—O5	0.9 (3)
N2—C3—C4—C5	0.7 (2)	C3—C4—C5—N1	0.3 (2)
C3A—C3—C4—C5	178.7 (2)	C4A—C4—C5—N1	−179.25 (18)
N2—C3—C4—C4A	−179.80 (19)		

### Hydrogen-bond geometry (Å, °)

**Table:**

*D*—H···*A*	*D*—H	H···*A*	*D*···*A*	*D*—H···*A*
N1—H1···O5^i^	0.91 (2)	1.82 (2)	2.730 (2)	175 (2)
N2—H2···O5^ii^	0.96 (2)	1.75 (2)	2.693 (2)	168 (2)

Symmetry codes: (i) −*x*, −*y*, −*z*+1; (ii) *x*, −*y*+1/2, *z*+1/2.
